# Beneficial Effects of Tomato Juice Fermented by *Lactobacillus Plantarum* and *Lactobacillus Casei*: Antioxidation, Antimicrobial Effect, and Volatile Profiles

**DOI:** 10.3390/molecules23092366

**Published:** 2018-09-16

**Authors:** Yiyun Liu, Haiming Chen, Wenxue Chen, Quipping Zhong, Guanfei Zhang, Weijun Chen

**Affiliations:** College of Food Science and Technology, Hainan University, Haikou 570228, China; 17889841410@163.com (Y.L.); hmchen168@126.com (H.C.); hnchwx@163.com (W.C.); hainufood88@163.com (Q.Z.); zgfei1021@126.com (G.Z.)

**Keywords:** tomato juice, *Lactobacillus plantarum*, *Lactobacillus casei*, antioxidant activity, *Escherichia coli* flora, volatile compounds

## Abstract

Tomato juice was fermented by *Lactobacillus plantarum* and *Lactobacillus casei* to produce an innovative high-bioactivity probiotic beverage. The levels of lycopene, total carotenoids, ascorbic acid, total phenolic and volatile compounds, 1,1-diphenyl-2-picrylhydrazyl (DPPH), 2,2’-azinobis-3-ethylbenzotiazo-line-6-sulfonic acid (ABTS) radical scavenging capacities, ferric reducing antioxidant power (FRAP), and *Escherichia coli* flora, as well as the inhibition of copper-induced human low-density lipoproteins (LDL)-cholesterol oxidation assays, were measured. The results revealed that the ABTS and DPPH inhibition values, as well as the FRAP and total phenolic content, were significantly increased. LDL-cholesterol oxidation was markedly delayed after the addition of the fermented juice. The in vitro inhibitory effects of *Escherichia coli* flora were substantially increased after being fermented with *Lactobacillus plantarum* and *Lactobacillus casei*. The results associated with the volatile compounds indicated that fermentation with *Lactobacillus plantarum* and *Lactobacillus casei* is a meaningful strategy for modifying flavors.

## 1. Introduction

Tomato is one of the major vegetables widely consumed worldwide. The health benefits of the tomato are attributed to its abundance in antioxidative components, including lycopene, phenolics, β-carotene, lutein and ascorbic acid [1,2,3,4,5,6,7].

Various studies have demonstrated the benefits of probiotics to human health [8]. Probiotics are routinely utilized in the manufacturing of fermented food products because they enhance the quality, safety, and sensory value of new functional foods [9]. *Lactobacillus casei* and *Lactobacillus plantarum* are two major probiotics that significantly enhance the antioxidative and antimicrobial activities of various foods because of their biosurfactants. All analyzed probiotic organisms that produce exopolysaccharides (EPS) under laboratory conditions have strong antibacterial abilities and scavenging activities [10]. Furthermore, fermentation is a well-known strategy for intensifying flavors.

This study aimed to investigate the feasibility of improving the antioxidant activity, antimicrobial ability and flavor of fermented tomato juice by *L. plantarum* and *L. casei*. In addition, the content of lycopene, total carotenoids, ascorbic acid, total phenolic and volatile compounds, 1,1-diphenyl-2-picrylhydrazyl (DPPH) and 2,2’-azinobis-3-ethylbenzotiazo-line-6-sulfonic acid (ABTS) radical scavenging capacities, ferric reducing antioxidant power (FRAP), and antimicrobial ability (*Escherichia coli* flora), as well as the inhibition of copper-induced human low-density lipoproteins (LDL)-cholesterol oxidation assays, were measured to determine whether fermentation by *L. plantarum* and *L. casei* can improve the bioactivity of tomato juice.

## 2. Results and Discussion

### 2.1. Juice Yield, Solid Content and pH Value

**Figure 1:**
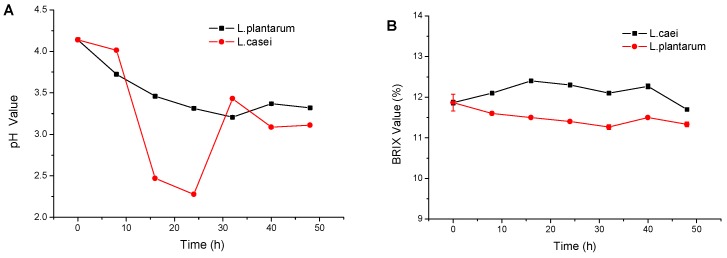
Changes of pH (**A**) and Brix values (**B**) of tomato juice during fermentation.

Figure 1 shows the changes in juice yield, solid content, and pH value. Approximately 6656 g of juice was obtained after processing 7446 g of tomatoes. The juice yield was 89.4%. The solid content was initially 4.5% and was adjusted to 12.2% for fermentation. No significant change was caused by fermentation regardless of bacterial strain (*L. casei* or *L. plantarum*) throughout the fermentation; by contrast, the pH value notably decreased. For *L. plantarum*, the pH displayed a downward trend until 3.20 (32 h) and then increased to 3.32 (48 h). For *L. casei*, the pH sharply dropped to 2.27 (24 h), increased to 3.43 (32 h), and was ultimately stable at 3.11 (48 h). The pH of the tomato juice fermented by *L. casei* at 32 h notably increased. *L. casei* can survive at such a pH value. However, α-tomatine can be released during the fermentation process, and a greater amount of acid corresponds to a higher α-tomatine content. This phenomenon may be the reason why the pH value increased in both fermented juices after fermentation for a few hours. For *L. casei*, a previous study has suggested that biogenic amine would be produced [11].

### 2.2. Chemical Characteristics of the Tomato Juice

**Figure 2:**
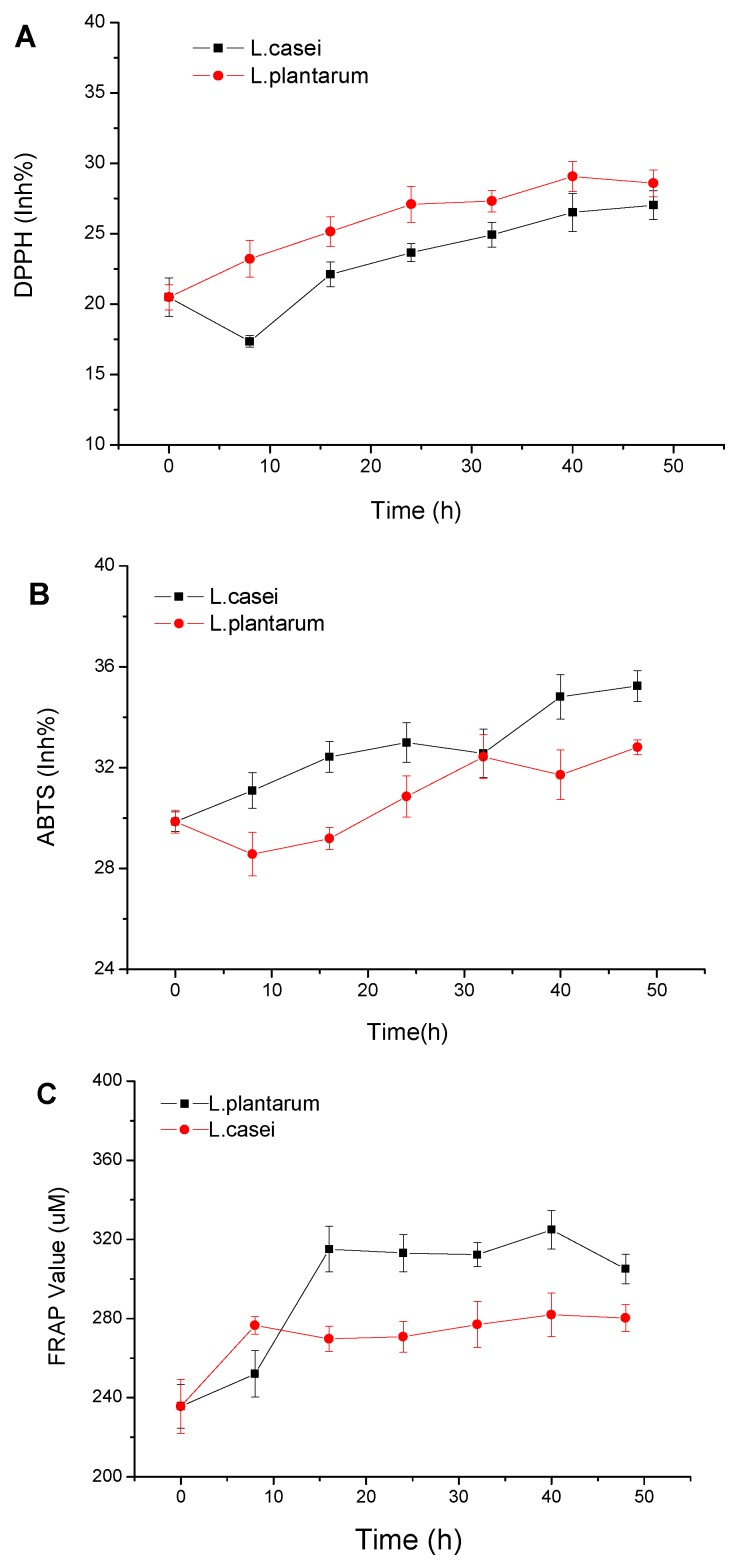
Changes of DPPH (**A**); ABTS (**B**) and FRAP (**C**) values of tomato juice during fermentation.

Figure 2 illustrates the free radical scavenging activity assay of the tomato juice. The antioxidant activities of *L. plantarum* and *L. casei* were evaluated from 0 to 48 h of fermentation in terms of ABTS assay, DPPH activity, FRAP, and ascorbic acid. The DPPH radical scavenging activity in the tomato juice significantly (*p* < 0.05) grew in the two groups, from 20.48% to 27.02% for *L. casei*, and from 20.48% to 28.57% for *L. plantarum*. The same trend was observed in the FRAP assay. Under significance analysis (*p* < 0.05), the data revealed a remarkable increase from 235.67 to 305.02 uM for *L. casei,* and from 235.67 to 280.27 uM for *L. plantarum*.

### 2.3. Copper-Induced Human LDL-Cholesterol Oxidation

**Figure 3:**
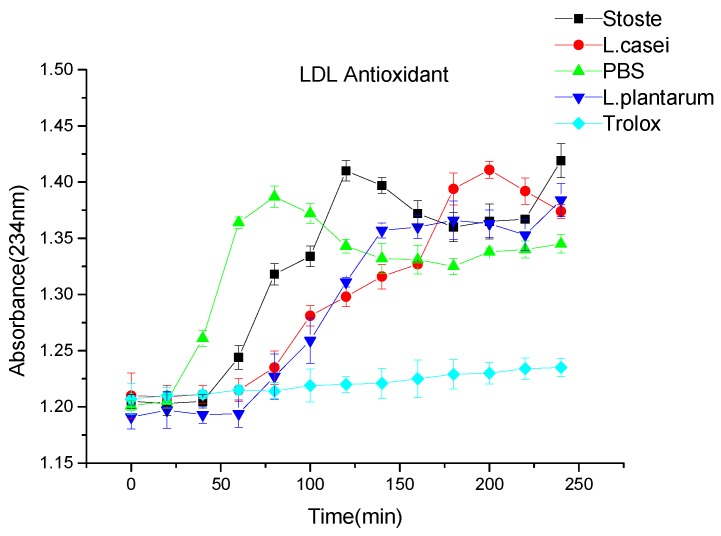
Changes of low-density lipoproteins (LDL)-cholesterol antioxidant capacity of tomato juice during fermentation.

LDL-cholesterol (Figure 3) has been found to be unstable, and the addition of antioxidants, such as Trolox, prevents the oxidation of LDL and plasma [12]; our results agree with these findings. As shown in Figure 3, the lag time was 40 min in the blank group and more than 240 min in the control (Trolox). Figure 3 shows that the antioxidant capacity of fermented tomato juice (lag time: 80 min) was higher than that of fresh tomato juice (60 min) with respect to LDL oxidation. This result was consistent with a previous finding wherein oxidized human LDL-cholesterol induced by copper was inhibited by phenolics [13]. Moreover, the inhibitory effect was correlated with the phenolic content [14]. Numerous studies have indicated that some Lactic acid bacteria (LAB) greatly inhibits the LDL oxidative effects, including those of *Lactobacillus* (*L.) delbrueckii*, *Bifidobacterium* (*B.) longum*, and *Streptococcus thermopillus* [15,16,17]. In addition, tomato juice can increase the resistance of LDL particles from the copper-induced formation of oxidized phospholipids [18], which agreed with our results for *L. casei* and *L. plantarum*.

### 2.4. Gut Bacteria Inhibition

**Figure 4:**
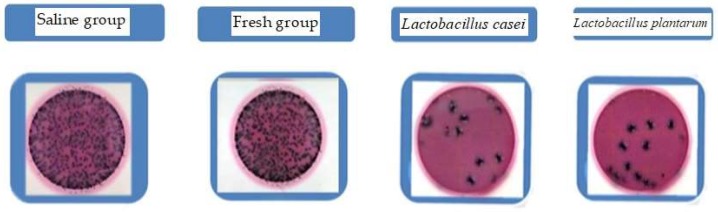
The bactericidal capacity of fresh and fermented tomato juice.

As shown in Figure 4, the saline and fresh tomato juice groups (10^-2^ times diluent) formed quantities of intestinal flora (including *E. coli*). By contrast, the inhibitory effect of *E. coli* was displayed well after the fermented juice was poured, regardless of the probiotic used (*L. casei* or *L. plantarum*). No marked differences in intestinal flora were observed among the saline, fresh tomato juice, and fermented juice groups. After the fermented juice was poured, the inhibitory effect displayed was possibly due to the following reasons. For the juice fermented by *L. casei*, LiN333 was found to effectively act against *Escherichia*. This activity might be due to some unknown characteristics of the outer membrane of *L. casei* [19]. Moreover, *L. plantarum* released an effective compound responsible for the important effect of disrupting the *E. coli* plasma membrane [20]. Other studies have also illustrated that three strains of *L. plantarum* from Balkan cheeses (CLP1, CLP2, and CLP3) exhibit high antibacterial activities that can be harnessed to control pediatric diarrhea, which is caused by the pathogenic strains of *E. coli* [21]. In addition, *L. plantarum* could affect the *E. coli*-induced passage of mannitol across the intestinal wall [22].

### 2.5. Changes of Total Phenolics during Fermentation

Table 1 shows that the total phenolics in the tomato juice fluctuated slightly but generally increased during fermentation with *L. casei*. The total phenolics initially peaked at 204.94 ug GAE/mL after 8 h, from 166.05 ug GAE/mL initially, followed by a mild downward trend for up to 24 h (to 153.65 ug GAE/mL), and then by another increase until the end (to 195.55 ug GAE/mL). The *L. plantarum* group did not change obviously (Table 1), however, it displayed the same trend. The total phenolics initially peaked at 167.42 ug GAE/mL after 8 h, from 166.05 ug GAE/mL initially, followed by a mild downward trend for up to 32 h (to 152.34 ug GAE/mL), and then by another increase until the end (to 170.04 ug GAE/mL). Phenolic compounds contribute highly to the antioxidative capacity of many plants [23], and this may explain the elevated antioxidative activity in fermented tomato juice. After fermentation by *L. casei* and *L. plantarum* [24], the ABTS, DPPH, and FRAP levels markedly increased, and these increases may be due to various reasons. All of the analyzed probiotic organisms produced EPS under laboratory conditions; moreover, probiotic organisms have strong antibacterial ability and strong scavenging activity [10]. For *L. plantarum*, r-EPS1 and r-EPS2 represent potent antioxidative activities for hydroxyl and DPPH radical scavenging and for reducing power assays [25]. For *L. casei*, the bio-surfactants from the strains exhibit considerable antioxidant and antiproliferative potencies.

### 2.6. Changes in Lycopene, Total Carotenoids, and Ascorbic Acid

As represented by Table 1, lycopene and total carotenoids levels showed similar tendencies regardless of the bacterial strain used for fermentation (*L. casei* or *L. plantarum*). Moreover, lycopene and total carotenoids levels significantly increased after fermentation with *L. casei* (from 191.51 and 153.55 ug/mL, to 202.41 and 158.56 ug/mL, respectively). By contrast, in Table 1, the tomato juice fermented by *L. plantarum* fluctuated slightly but generally decreased (from 191.51 and 153.55 ug/mL, to 161.39 and 142.27 ug/mL, respectively). In comparison, the data did not significantly vary for ascorbic acid (for *L. casei* and *L. plantarum*).

### 2.7. Volatile Compositions of Tomato Juice before and after Fermentation

Various volatile compounds, including alcohols, hydrocarbons, acids, esters, aldehydes, and ketones, were detected and identified by GC-MS in fresh and fermented (*L. casei* and *L. plantarum*) tomato juices, as shown in Table 2.

Table 2 shows that after fermentation with *L. casei* and *L. plantarum*, the volatile compounds exhibited kinetic change trends: some increased, some were unchanged, some decreased, and some were similar. Moreover, alcohols, acids, and ketones increased, and hydrocarbons, aldehydes, and esters decreased. Nonetheless, new products were produced differently.

Alcohols were the most abundant group among the volatile compounds in fresh and fermented tomato juices, with total relative peak areas (RPA) of 49.26% (fresh), 59.92% (*L. casei*), and 49.7% (*L. plantarum*). Moreover, most alcohols decreased after being fermented by *L. plantarum*, except for 4-methyl-1-hexanol (from 0.09% to 0.21%). A different situation was observed for the tomato juice fermented by *L. casei*, in which nearly half increased and the other half decreased. For both strains, quantities of alcohol were produced, resulting in increased total alcohol content.

In addition, *Lactobacillus* fermentation resulted in increased fatty acid concentration in the fermented tomato juice (Table 2). The newly produced ammonium acetate (10.25%) was responsible for this change in the tomato juice fermented by *L. casei*. Moreover, acetic acid (from 2.78% to 26.72%) accounted for this change in the tomato juice fermented by *L. plantarum*.

Ketones increased from 0.51% (initially) to 14.84% (*L. casei*) and 12.07% (*L. plantarum*) after fermentation. Many of the substances came out, especially for 6-methyl-5-hepten-2-one, leading to these notable conversions (10.1% (*L. casei*) and 8.71% (*L. plantarum*)).

Volatile hydrocarbons, which are another major group of volatile compounds, significantly decreased from 15.12% (initially) to 4.24% (*L. casei*) and 2.26% (*L. plantarum*) after fermentation. Moreover, all but 2-, 6-, 10-, and 14-tetramethyl-hexadecane increased in both fermented juices. For the juice fermented by *L. casei*, 1, 3-dimethyl-benzene increased as well. However, similar to the alcohol group, new products were formed. 

The aldehyde categories and levels of fresh and fermented tomato juices are presented in Table 2. After *Lactobacillus* fermentation, aldehyde concentration significantly decreased from 25.55% to 0.21% (*L. casei*) and 1.89% (*L. plantarum*). Apart from the newly produced E-2-Undecenal, the others, after fermentation by *L. casei*, decreased. By contrast, all concentrations, including those of the newly appeared (E)-2-Octenal and (Z)-3, 7-dimethyl -2,6-Octadienal, decreased after fermentation by *L. plantarum*.

Esters are important components that confer fermented juices with fruity or floral flavors, which form during fermentation. As shown in Table 2, *Lactobacillus* fermentation slightly reduced the total esters (from 2.28% (fresh) to 1.99% (*L. casei*) and 0.37% (*L. plantarum*)) mainly due to the disappearance of ethyl acetate (1.87%, fresh). However, the newly formed methyl undecyl ether (0.34%) and ethyl tridecanoate (0.46%) after fermentation by *L. casei* did not change this situation. After fermentation by *L. plantarum*, the levels of dodecanoic acid, methyl ester (0.05%), tetradecanoic acid, and ethyl (0.03%) ester were not converted.

These changes were probably due to the activity and variety of enzymes related to *L. plantarum* and chemical changes of *L. casei* [26]. Alcohols are important in tomato juice flavor. A new class of substances was formed and showed different aromatic components due to various *Lactobacillus* strains. Esters are aromatic compounds, which appeared as new substances in the fermented tomato juice. Apart from alcohols and esters, other components also appeared in our study, and possibly contributed to the antioxidative and antimicrobial properties of the tomato juice [27].

## 3. Materials and Methods 

### 3.1. Materials

Active dry *L. plantarum* and *L. casei* were obtained from Lallemand Inc. (Montreal, Quebec, Canada). Tomatoes were bought from the local market. DPPH, ABTS, 6-hydroxy-2,5,7,8-tetramethylchroman-2-carboxylic acid (Trolox), and 2,4,6-tris(2-pyridyl)-s-triazine (TPTZ) were acquired from Tokyo Chemical Industry Co., Ltd., (TCI, Tokyo, Japan). Low-density lipoprotein was purchased from ProSpec Inc., (Rehovot, Israel). Folin-Ciocalteu phenol and neocuproine reagents were purchased from Merck Corporation (Merck, Darmstadt, Germany). General anaerobic medium and eosin methylene blue were purchased from Hopebio Corporation (Qingdao, China). All other chemicals were of analytical grade unless otherwise stated. 

### 3.2. Juice Preparation

Fresh and ripe tomatoes were washed and rid of the ends. The juice was extracted from the tomatoes using a Breville BBl605XL Hemisphere blender (Breville, Sydney, Australia) and a relatively fine tomato juice was obtained by using a plastic filter (200 sieve pore). Approximately 500 mL of the juice was sealed in a plastic bag and refrigerated after pasteurization (90 °C, 30 s). The total soluble solid content of the juice was adjusted from 4.6 °Brix to 12.4 °Brix with glucose (final concentration of 5%, *w*/*w*) and then sucrose. Afterward, the fruit juice blends were pasteurized using hot water at 90 °C for 10 min and cooled under running water. Lastly, we sealed the tomato juice inside sterile plastic bags that were refrigerated at −80 °C.

### 3.3. Microbial Cultivation

#### 3.3.1. Open Ampoule 

A degreasing cotton that had been soaked in 75% alcohol was rubbed along the neck of the ampoule, the top of which was then flame-heated. Sterile water was then dropped to make it break, and tweezers were used to knock down the top.

#### 3.3.2. Strain Recovery Training

Aseptic suction drains were used to suction 0.5 mL MRS and the skim milk liquid medium, which were then dripped into the *L. plantarum* and *L. casei* ampoule pipes under gentle shaking. The samples were then fully dissolved in slurry freeze-dried bacteria. All bacterial suspensions were then absorbed by inoculation in MRS and the skim milk medium. The samples were incubated at a constant anaerobic temperature of 37 °C for 48 h.

#### 3.3.3. Strain-Activated Cultivation 

The *L. plantarum* and *L. casei* were collected from the constant anaerobic temperature incubator and transferred into the MRS and skim milk medium (5% proportion). The samples were incubated at a constant anaerobic temperature of 37 °C for 24 h and then refrigerated at 4 °C. 

### 3.4. Pretreatment Method

To determine the water-soluble antioxidant indicator, a pretreatment reagent was prepared at the beginning.
Use a pipette to suck a 5 mL sample into the centrifugal tube↓20 mL 80% ethyl alcohol↓Ultrasound bath at 40 °C for 30 min↓Pour 25 mL of supernatant liquor into the volumetric flask until constant volume↓Seal the tube to avoid light preservation (4 °C)


### 3.5. Chemical Characteristics of the Fruit Juice Blends

#### 3.5.1. Determination of pH and Total Soluble Solids (°Brix)

The total soluble solids (°Brix) of the juice were measured using a refractometer (ATAGO, Tokyo, Japan) that had been previously adjusted to zero with distilled water. A pH meter (Hanna Instrument, Poroa de Varzim, Portugal) that had been previously calibrated with buffer solutions (4.86 and 9.18) was used to determine the pH of the samples.

#### 3.5.2. Determination of DPPH Radical Scavenging Activity

A DPPH assay was performed according to a previously described method with slight modifications [28]. The DPPH solution was diluted with ethanol to achieve an absorbance of 1.2–1.3 at 517 nm. For the DPPH assay, 3.5 mL DPPH solution was added to 0.5 mL of samples. The solution was mixed well and incubated in the dark at 30 °C for 45 min. The DPPH absorbance was read at 517 nm by using a TU-1810 spectrophotometer (Persee, Beijing, China). The DPPH radical scavenging capacities of the samples were calculated using the following equation:

DPPH radical scavenging capacity (%) = [(A0−A1)/A0] × 100%

where A0 is the absorbance of the control (ethanol) and A1 is the absorbance of the sample.

#### 3.5.3. Determination of ABTS· Radical Scavenging Activity

The ABTS**·** radical scavenging activity of the tomato juice was measured using a previously described procedure [29]. ABTS stock solution (2 mL of ABTS stock solution (0.01 M) added to 58 mL phosphate buffer saline, pH 6.9) was incubated for 12 h and diluted with ethanol to achieve an absorbance of 0.70 (± 0.02) at 734 nm and equilibrated at 30 °C, and the absorbance was read at 734 nm.

The ABTS**·** radical scavenging capacities of the samples were calculated using the following equation:

ABTS radical scavenging capacity (%) = [(A0−A1)/A0] × 100%

where A0 is the absorbance of the control (ethanol) and A1 is the absorbance of the sample.

#### 3.5.4. FRAP Assay

A FRAP assay was performed according to a previously described method [30]. The FRAP reagent, which consisted of acetate buffer solution (0.3 M, pH 3.6), FeCl_3_·6H_2_O solution (20 mM), and TPTZ solution (10 mM in 40 mM HCl) (10:1:1, v/v/v), was added to 0.5 mL of extraction solution and kept in the dark for 30 min. The absorbance of the mixture was detected at 593 nm by using a TU-1810 spectrophotometer (Persee, Beijing, China). FeSO_4_·7H_2_O was used as the standard, and the results were expressed as μM FeSO_4_·7H_2_O equivalents.

#### 3.5.5. Determination of Total Phenolic Compounds (TPC)

The total phenolic compounds in the tomato juice were determined using the Folin-Ciocalteu reagent, with gallic acid as the standard according to a previously described method with slight modifications [31]. The sample (0.5 mL) was diluted with deionized water (3.3 mL) and added with Folin−Ciocalteu reagent (0.2 mL). The sample was placed in the dark at room temperature for 5 min. Afterward, 10% Na_2_CO_3_ was transfused with 1.3 mL saturated solution, and then the mixture was left in the dark for 1.5 h. The absorbance of the mixture was evaluated by contrasting with a blank reagent at 765 nm by using a TU-1810 spectrophotometer (Persee, Beijing, China). Gallic acid (0–8.2 μg/m) was used as the standard comparison. The total phenolic compounds were expressed as gallic acid equivalents per milliliter of sample.

#### 3.5.6. Determination of Lycopene and Total Carotenoids

The lycopene and total carotenoids contents were determined using spectrophotometry according to the method of Bamidele and Fasogbon [23]. A mixture of hexane-acetonitrile-ethanol [50:25:25 (*v/v/v*)] was used to extract the lycopene and total carotene. In briefly, an approximately 1-mL sample was extracted from the 50-mL solvent mixture. The mixture was placed in the dark to extract the carotenoids with stirring (15 min) on a magnetic stirring plate. Distilled water (3 mL) was added to the mixture, stirred for another 5 min. Then the mixture was allowed to settle until the phase separation (~5 min). The contents of lycopene and total carotenoids were obtained by measuring the absorbance of the filtered hydrophobic phase at 503 and 450 nm, respectively. Hexane was used as the blank.

#### 3.5.7. Ascorbic Acid Content (AA)

The AOAC (2005) method was used to determine the ascorbic acid content [32]. A pipette was used to transfuse 20 mL of tomato juice to produce up to 50 mL with oxalic acid (0.1 M) and then filtered. The filtrate (5 mL) was dispensed into a beaker with a pipette and titrated with standardized 2, 6-dichlorophenol indophenol dye. The colored solution changed from orange to pink to mark the endpoint of the titration. The procedure was repeated 3 times. Ascorbic acid was used as the standard comparison.

#### 3.5.8. Copper-Induced Human LDL-Cholesterol Oxidation

The in vitro LDL-cholesterol oxidation procedure used was conducted according to a previously described method [33] with slight modifications. The LDL was diluted with PBS (10 mM, pH 7.4) to a concentration of 100 μg/mL, and oxidation was initiated by Cu^2+^ (final concentration: 5 μM). The oxidation kinetics were measured based on the change in absorbance at 234 nm by using a TU-1810 spectrophotometer (Persee, Beijing, China), in which the absorbance was monitored every 20 min at 37 °C for 4 h. The absorbance–time curve can be divided into three states: lag, propagation, and decomposition phases. The interception point of the tangents of the lag and propagation phases was defined as the lag time. We chose a slope that demonstrated antioxidant activity (a higher slope corresponded to a worse antioxidant activity). For the assay, LDL (4 mL) was added to fermented tomato juice (final concentration of 0.05 mL/mL), tomato juice (final concentration of 0.05 mL/mL), and Trolox solution (final concentration of 0.005 mg/mL) and incubated at 37 °C for 15 min. Afterward, peroxidation was initiated with 5 μM CuSO_4_ solution.

#### 3.5.9. Effects of Fermented Fruit and Vegetable on the Intestinal Flora

All groups used the same saline dilute stool suspension (1:10) that was pipetted (1 mL) to the initial test tube. Afterward, concentration gradient dilution was performed to determine the appropriate concentration of the saline dilute stool suspension (pH value was adjusted at 6.5–7.5). Then, 1 mL diluted liquid was transferred from the test tube with an appropriate concentration to 3M™ Petrifilm™ *E. coli*/Coliform Count (EC) Plates (Minnesota Mining Manufacturing (Shanghai) International Trade Co., Ltd) [34]. As shown in Figure 4, the red dot with the bubble is the intestinal flora, and the blue one is *E. coli*.

#### 3.5.10. Determination of Volatile Compounds

The volatile compounds were analyzed using headspace-solid phase microextraction sampling combined with gas chromatography–mass spectrometry (GC-MS) according to a previously described method [9]. Approximately 50% of the sample volume was added with 0.30 g/mL NaCl in a headspace glass. Carboxen/Polydimethylsiloxane (CAR/PDMS) (75 µm) (Sigma-Aldrich, Saint Louis, MO, USA) fiber was used for extraction for 40 min at 50 °C, followed by desorption for 3 min at 230 °C. The data were collected using GC solution software (Shimadzu, Kyoto, Japan). Identification was achieved by matching the mass spectrum against the National Institute of Standards and Technology (NIST) 11 libraries and confirmed with the linear retention index values.

## 4. Conclusions 

This study demonstrated that tomato juice can be fermented with selected LAB (*L. casei* and *L. plantarum*) as probiotic microorganisms in order to improve its antioxidative effects, regardless of the anti-*E*. *coli* and aroma effects. Therefore, fermenting with *L. casei* and *L. plantarum* can make tomato juice a potential probiotic product that is highly functional and health enhancing.

## Figures and Tables

**Table 1:** Changes of tomato juice during fermentation by *L. casei* ^c^ and *L. plantarum ^1,^*^d*.*^

Hours of Fermentation	TPC ^2,c^	Lycopene ^c^	β-Carotinoid ^c^	Ascorbic Acid ^c^	TPC ^2,d^	Lycopene^d^	Total Carotenoids ^d^	Ascorbic Acid ^d^
	(ug GAE/mL)	(ug/mL)	(ug/mL)	(ug/mL)	(ug GAE/mL)	(ug/mL)	(ug/mL)	(ug/mL)
0	166.05 ± 2.12 ^d^	191.51 ± 2.14 ^c^	153.55 ± 0.81 ^d^	9.77 ± 2.97 ^a,b^	166.05 ± 9.25 ^a^	191.51 ± 8.75 ^a^	153.55 ± 2.62 ^a^	11.65 ± 1.82 ^a^
8	204.94 ± 4.75 ^a^	210.34 ± 3.96 ^a^	161.32 ± 1.42 ^a^	7.94 ± 0.22 ^b^	167.42 ± 10.97 ^a,b^	182.59 ± 1.91 ^a^	149.67 ± 0.71 ^a,b^	12.94 ± 4.98 ^a^
16	184.16 ± 6.47 ^b,c^	193.69 ± 3.96 ^c^	154.47 ± 1.59 ^c,d^	7.75 ± 0.77 ^b^	165.41 ± 5.81 ^a,b^	179.23 ± 3.57 ^a,b^	148.24 ± 1.23 ^a,b^	11.38 ± 1.42 ^a^
24	153.65 ± 8.19 ^d^	200.63 ± 2.48 ^b^	157.03 ± 0.99 ^b,c^	8.56 ± 0.93 ^b^	161.69 ± 4.61 ^b^	188.14 ± 4.49 ^a,b^	152.12 ± 2.18 ^a^	12.06 ± 1.23 ^a^
32	168.07 ± 7.27 ^c^^,d^	202.21 ± 5.45 ^b^	158.46 ± 2.73 ^a,b^	7.86 ± 0.12 ^b^	152.34 ± 8.37 ^b^	192.7 ± 8.92 ^a,b^	154.17 ± 2.73 ^a^	14.09 ± 1.38 ^a^
40	182.82 ± 10.07 ^b,c^	197.46 ± 2.06 ^b,c^	156.41 ± 0.99b ^c,d^	7.79 ± 0.12 ^b^	169.03 ± 2.09 ^a,b^	179.62 ± 4.22 ^a,b^	148.64 ± 1.85 ^a,b^	13.08 ± 1.66 ^a^
48	195.55 ± 1.59 ^a,b^	202.41 ± 3.96 ^b^	158.56 ± 1.87 ^a,b^	11.11 ± 1.02 ^a^	170.04 ± 6.06 ^a,b^	161.39 ± 5.95 ^b^	142.27 ± 2.77 ^b^	12.13 ± 0.82 ^a^

^1^ Values are expressed as mean ± SD; values in the same column followed by different letters (a–d) are statistically different at *p* < 0.05; ^2^ TPC, total phenolic content.

**Table 2:** Major volatile compounds (peak area × 10^6^) and relative peak area (RPA) in fermented tomato juice.

Category	Volatile Compounds	Tomato Juice		Fermented Tomato Juice (*L. casei*)		Fermented Tomato Juice (*L. Plantarum*)	
		Peak Area	RPA (%)	Peak Area	RPA (%)	Peak Area	RPA (%)
Alcohols	Ethanol	45.04 ± 1	3.83	36.8 ± 0.16	4.33	16.95 ± 0.42	1.61
	1-Butanol	2.07 ± 0.03	0.18	ND	0	ND	0
	1-Butanol, 3-methyl-	58.93 ± 0.35	5	43.8 ± 0.08	5.15	116.16 ± 0.73	11.04
	1-Pentanol	68.14 ± 1.35	5.79	54.16 ± 0.9	6.37	27.58 ± 0.6	2.62
	2-Tridecen-1-ol, (*E*)-	16.65 ± 0.75	1.41	ND	0	ND^*^	0
	1-Penten-3-ol	15.95 ± 0.77	1.35	4.69 ± 0.21	0.55	ND	0
	(S)-(+)-3-Methyl-1-pentanol	4.79 ± 0.1	0.41	ND	0	ND	0
	1-Hexanol	200.44 ± 0.4	17.02	157.42 ± 6.08	18.51	102.69 ± 0.83	9.76
	3-Hexen-1-ol, (*Z*)-	43.93 ± 0.97	3.73	29.85 ± 0.28	3.51	16.14 ± 0.08	1.53
	2-Hepten-1-ol, (*E*)-	7.05 ± 0.1	0.6	3.43 ± 0	0.4	ND	0
	1-Hexanol, 4-methyl-, (*S*)-	1.1 ± 0.04	0.09	0.32 ± 0.01	0.04	2.17 ± 0	0.21
	1-Octen-3-ol	9.39 ± 0.13	0.8	4.19 ± 0.16	0.49	ND	0
	1-Heptanol	4.6 ± 0.06	0.39	4.44 ± 0.15	0.52	3.01 ± 0.07	0.29
	1-Pentanol, 2-methyl-	0.44 ± 0.01	0.04	ND	0	ND	0
	6-Hepten-1-ol, 2-methyl-	2.17 ± 0.1	0.18	3.26 ± 0.14	0.38	ND	0
	2-Propyl-1-pentanol	1.67 ± 0.04	0.14	ND	0	ND	0
	1-Octanol	6.82 ± 0.09	0.58	9.93 ± 0.22	1.17	5.4 ± 0.07	0.51
	Bicyclo[2.2.1]heptan-2-ol, 1,3,3-trimethyl-	2.35 ± 0.08	0.2	ND	0	ND	0
	5-Isopropenyl-2-methyl-7-oxabicyclo[4.1.0]heptan-2-ol	0.48 ± 0.02	0.04	ND	0	ND	0
	1-Methylcycloheptanol	0.65 ± 0	0.05	0.72 ± 0.04	0.08	ND	0
	2-Octen-1-ol, (*E*)-	5.47 ± 0.11	0.46	ND	0	ND	0
	Phenylethyl Alcohol	0.94 ± 0.05	0.08	1.29 ± 0.02	0.15	ND	0
	2-Hexen-1-ol, (*Z*)-	81.11 ± 2.17	6.89	56.7 ± 0.27	6.67	36.09 ± 1.12	3.43
	.alpha.-Terpineol	ND	0	6.16 ± 0.02	0.72	ND	0
	3-Nonen-1-ol, (*Z*)-	ND	0	0.59 ± 0.01	0.07	ND	0
	trans-2-Undecen-1-ol	ND	0	6.92 ± 0.16	0.81	ND	0
	2-Propyl-1-pentanol	ND	0	6.48 ± 0.28	0.76	ND	0
	1-Pentanol, 3-methyl-	ND	0	10.19 ± 0.12	1.2	46.72 ± 1.3	4.44
	2-Penten-1-ol, (*Z*)-	ND	0	3.27 ± 0.08	0.38	0.93 ± 0.05	0.09
	3-Hexen-1-ol, acetate, (*Z*)-	ND	0	6.07 ± 0.13	0.71	ND	0
	2-Penten-1-ol, (*Z*)-	ND	0	13.74 ± 0.16	1.62	ND	0
	3-Buten-1-ol, 3-methyl-	ND	0	7.24 ± 0.31	0.85	7.49 ± 0.36	0.71
	4-Hexen-1-ol	ND	0	7.09 ± 0.01	0.83	ND	0
	2-Nonanol	ND	0	4.12 ± 0	0.49	ND	0
	trans-(2-Ethylcyclopentyl)methanol	ND	0	3.21 ± 0.06	0.38	ND	0
	cis-9-Tetradecen-1-ol	ND	0	0.59 ± 0.02	0.07	ND	0
	2-Furanmethanol	ND	0	1 ± 0.05	0.12	ND	0
	2-Nonen-1-ol, (*E*)-	ND	0	0.22 ± 0.01	0.03	ND	0
	Geraniol	ND	0	1.77 ± 0.06	0.21	ND	0
	5,9-Undecadien-2-ol, 6,10-dimethyl-	ND	0	1.07 ± 0.03	0.13	ND	0
	Bicyclo[3.1.1]hept-2-ene-2-methanol, 6,6-dimethyl-	ND	0	0.42 ± 0.02	0.05	ND	0
	1,6-Octadien-3-ol, 3,7-dimethyl-	ND	0	18.45 ± 0.25	2.17	14.23 ± 0.27	1.35
	Acetoin	ND	0	ND	0	24.71 ± 0.64	2.35
	3-Hexen-1-ol, (*E*)-	ND	0	ND	0	4.15 ± 0.12	0.39
	1-Hexanol, 2-ethyl-	ND	0	ND	0	41.16 ± 1.18	3.91
	3-Furanmethanol	ND	0	ND	0	1.12 ± 0.05	0.11
	Phenylethyl Alcohol	ND	0	ND	0	4.51 ± 0.17	0.43
	2-Buten-1-ol, 3-methyl-	ND	0	ND	0	12.62 ± 0.29	1.2
	1-Pentanol, 4-methyl-	ND	0	ND	0	9.46 ± 0.37	0.9
	3-Hexen-1-ol, acetate, (*E*)-	ND	0	ND	0	2.02 ± 0.05	0.19
	6-Hepten-1-ol, 2-methyl-	ND	0	ND	0	16.29 ± 0.01	1.55
	1-Methylcycloheptanol	ND	0	ND	0	1.09 ± 0.04	0.1
	2-Tridecen-1-ol, (*E*)-	ND	0	ND	0	3.9 ± 0.04	0.37
	Citronellol	ND	0	ND	0	0.49 ± 0	0.05
	3-Nonen-1-ol, (*Z*)-	ND	0	ND	0	0.64 ± 0.02	0.06
	Geraniol	ND	0	ND	0	2.56 ± 0.02	0.24
	5,9-Undecadien-2-ol, 6,10-dimethyl-	ND	0	ND	0	2.74 ± 0.11	0.26
	subtotal	580.18	49.26	509.64	59.92	523.04	49.7
Hydrocarbons	Ethylbenzene	2.2 ± 0.07	0.19	ND	0	ND	0
	Benzene, 1,3-dimethyl-	0.53 ± 0	0.04	0.51 ± 0.01	0.06	ND	0
	p-Xylene	2.14 ± 0.05	0.18	ND	0	ND	0
	3-Carene	7.02 ± 0.17	0.6	ND	0	5.27 ± 0.04	0.5
	o-Xylene	0.87 ± 0.02	0.07	3.4 ± 0.02	0.4	ND	0
	Styrene	3.88 ± 0.1	0.33	1.15 ± 0.01	0.13	ND	0
	Cyclohexane, ethylidene-	2.29 ± 0.02	0.19	1.32 ± 0.01	0.16	ND	0
	Propylene Carbonate	20.37 ± 0.03	1.73	ND	0	ND	0
	Toluene	2.43 ± 0.01	0.21	ND	0	ND	0
	2-Octene, 2-methyl-6-methylene-	2.75 ± 0.02	0.23	ND	0	ND	0
	3-Hexen-1-ol, acetate, (*Z*)-	1.5 ± 0.01	0.13	ND	0	ND	0
	Pentane, 1-nitro-	24.29 ± 0.9	2.06	ND	0	ND	0
	Pentane, 2-nitro-	ND	0	14.43 ± 0.21	1.7	ND	0
	5-Hepten-2-one, 6-methyl-	90.79 ± 3.29	7.71	0 ± 0	0	ND	0
	Undecyne	0.35 ± 0.01	0.03	ND	0	ND	0
	Bicyclo[4.1.0]heptane, 7-(1-methylethylidene)-	1.21 ± 0.04	0.1	ND	0	ND	0
	Bicyclo[2.2.1]hept-2-ene, 1,7,7-trimethyl-	ND	0	0.84 ± 0.02	0.1	ND	0
	1,6-Octadien-3-ol, 3,7-dimethyl-	5.17 ± 0.22	0.44	ND	0	ND	0
	2-Furanmethanol	1.67 ± 0.08	0.14	ND	0	ND	0
	alpha.-Terpineol	6.34 ± 0.24	0.54	ND	0	ND	0
	Hexadecane, 2,6,10,14-tetramethyl-	2.39 ± 0.03	0.2	4.7 ± 0.23	0.55	7.08 ± 0.3	0.67
	1-Tetradecyne	ND	0	4.95 ± 0.09	0.58	ND	0
	1-Hexene, 3,3,5-trimethyl-	ND	0	2.52 ± 0.01	0.3	ND	0
	Heneicosane	ND	0	1.37 ± 0.07	0.16	ND	0
	4-Tridecene, (*Z*)-	ND	0	0.84 ± 0.03	0.1	ND	0
	d-Limonene	ND	0	ND	0	1.87 ± 0.05	0.18
	Butylated Hydroxytoluene	ND	0	ND	0	1.85 ± 0.04	0.18
	Benzene, 1-methoxy-4-methyl-	ND	0	ND	0	0.26 ± 0.01	0.02
	1-Hexene, 3,3-dimethyl-	ND	0	ND	0	1.99 ± 0.02	0.19
	Heneicosane	ND	0	ND	0	0.82 ± 0.01	0.08
	3-Nonen-5-yne, 4-ethyl-	ND	0	ND	0	4.09 ± 0.06	0.39
	3-Buten-2-one, 4-(2,6,6-trimethyl-1-cyclohexen-1-yl)-	ND	0	ND	0	2.4 ± 0.03	0.23
	subtotal	178.2	15.12	36.03	4.24	25.61	2.26
Aldehydes	Hexanal	175 ± 0.4	14.86	ND	0	7.7 ± 0.03	0.73
	2-Hexenal, (*E*)-	93.72 ± 3.31	7.96	ND	0	0.85 ± 0.02	0.08
	Octanal	1.4 ± 0.07	0.12	ND	0	ND	0
	Nonanal	8.5 ± 0.34	0.72	ND	0	3.55 ± 0.18	0.34
	Butanal, 3-methyl-	8.41 ± 0.12	0.71	ND	0	ND	0
	2,4-Hexadienal, (*E,E*)-	3.66 ± 0.06	0.31	ND	0	ND	0
	Decanal	0.87 ± 0.01	0.07	ND	0	0.28 ± 0.01	0.03
	2-Nonenal, (*E*)-	1.7 ± 0.08	0.14	0.8 ± 0.02	0.09	0.17 ± 0	0.02
	Benzene acetaldehyde	1.05 ± 0.02	0.09	ND	0	ND	0
	2,6-Octadienal, 3,7-dimethyl-, (*Z*)-	0.84 ± 0.01	0.07	ND	0	ND	0
	1-Cyclohexene-1-carboxaldehyde, 2,6,6-trimethyl-	2.45 ± 0.1	0.21	ND	0	ND	0
	2,6-Octadienal, 3,7-dimethyl-, (*E*)-	2.78 ± 0.09	0.24	ND	0	4.85 ± 0.09	0.46
	1H-Pyrrole-2-carboxaldehyde	0.57 ± 0.02	0.05	0.45 ± 0.02	0.05	0.78 ± 0.03	0.07
	2-Undecenal, E-	ND	0	0.58 ± 0.02	0.07	ND	0
	2-Octenal, (*E*)-	ND	0	ND	0	1.08 ± 0.05	0.1
	2,6-Octadienal, 3,7-dimethyl-, (*Z*)-	ND	0	ND	0	0.65 ± 0.01	0.06
	subtotal·	300.97	25.55	1.84	0.21	19.91	1.89
Ketones	Cyclohexanone, 2,2,6-trimethyl-	0.56 ± 0.02	0.05	ND	0	ND	0
	Acetone	3.67 ± 0.07	0.31	1.79 ± 0.05	0.21	ND	0
	4’-(Trifluoromethyl)acetophenone	0.51 ± 0.02	0.04	3.67 ± 0.02	0.43	ND	0
	trans-beta.-Ionone	1 ± 0.02	0.08	ND	0	ND	0
	3-Buten-2-one, 4-(2,2,6-trimethyl-7-oxabicyclo[4.1.0]hept-1-yl)-	0.38 ± 0.01	0.03	1.46 ± 0.03	0.17	0.47 ± 0.02	0.04
	4-Hydroxy-2-methylacetophenone	ND	0	0.25 ± 0.01	0.03	0.61 ± 0.03	0.06
	2-Undecanone	ND	0	0.25 ± 0	0.03	ND	0
	5-Hepten-2-one, 6-methyl-	ND	0	85.9 ± 3.47	10.1	91.68 ± 3.49	8.71
	2-Nonanone	ND	0	8.29 ± 0.4	0.98	1.47 ± 0.06	0.14
	2-Pentanone	ND	0	23.03 ± 0.25	2.71	ND	0
	2-Propanone, 1-hydroxy-	ND	0	0.47 ± 0.02	0.06	1.65 ± 0.08	0.16
	Acetoin	ND	0	0.99 ± 0.01	0.12	ND	0
	2-Cyclopenten-1-one, 3,4,4-trimethyl-	ND	0	ND	0	3.05 ± 0.06	0.29
	2-Heptanone	ND	0	ND	0	7.75 ± 0.2	0.91
	2,3-Butanedione	ND	0	ND	0	14.73 ± 0.24	1.4
	2-Heptanone	ND	0	ND	0	2.62 ± 0.04	0.25
	2H-Pyran-2-one, tetrahydro-	ND	0	ND	0	0.51 ± 0	0.05
	3,5,9-Undecatrien-2-one, 6,10-dimethyl-	ND	0	ND	0	0.2 ± 0	0.02
	2(4H)-Benzofuranone, 5,6,7,7a-tetrahydro-4,4,7a-trimethyl-, (*R*)-	ND	0	ND	0	0.37 ± 0	0.04
	subtotal	6.12	0.51	126.1	14.84	125.09	12.07
Acids	Acetic acid	32.74 ± 1.15	2.78	ND	0	281.29 ± 3.92	26.72
	Butanoic acid, 3-methyl-	7.41 ± 0.31	0.63	ND	0	22.28 ± 0.06	2.12
	Butanoic acid, anhydride	ND	0	0.3 ± 0	0.04	ND	0
	Hexanoic acid	3.84 ± 0.17	0.33	9.17 ± 0.44	1.08	12.85 ± 0.63	1.22
	Octanoic acid	ND	0	10.6 ± 0.46	1.25	0.36 ± 0	0.03
	Cyclohexylmethyl formate	ND	0	0.36 ± 0	0.04	ND	0
	Ammonium acetate	ND	0	87.11 ± 0.25	10.25	ND	0
	subtotal·	43.99	3.74	107.54	12.66	316.78	30.09
Esters	Ethyl Acetate	22 ± 0.36	1.87	ND	0	ND	0
	3-Nonen-5-yne, 4-ethyl-	4.86 ± 0.23	0.41	5.33 ± 0.2	0.63	ND	0
	Methyl undecyl ether	ND	0	2.86 ± 0.04	0.34	ND	0
	Ethyl tridecanoate	ND	0	3.93 ± 0.01	0.46	ND	0
	Acetic acid, butyl ester	ND	0	4.76 ± 0.13	0.56	3.05 ± 0.15	0.29
	Dodecanoic acid, methyl ester	ND	0	ND	0	0.49 ± 0	0.05
	Tetradecanoic acid, ethyl ester	ND	0	ND	0	0.32 ± 0.01	0.03
	subtotal	26.85	2.28	16.88	1.99	3.87	0.37
Others	Pyridine	1.01 ± 0.05	0.09	ND	0	ND	0
	2-Isobutylthiazole	7.72 ± 0.35	0.66	6.95 ± 0.13	0.82	ND	0
	Oxime-, methoxy-phenyl-_	24.13 ± 1.04	2.05	11.44 ± 0.3	1.35	ND	0
	1H-Isoindole, 3-methoxy-4,7-dimethyl-	0.63 ± 0.01	0.05	ND	0	ND	0
	Benzofuran, 2,3-dihydro-	1.11 ± 0.04	0.09	1.13 ± 0.01	0.13	17.49 ± 0.8	1.66
	Phenol, 2,4-bis(1,1-dimethylethyl)-	2.23 ± 0.11	0.19	1.75 ± 0.06	0.21	2.44 ± 0.07	0.23
	D-Limonene	ND	0	20.34 ± 0.69	2.39	ND	0
	Butanenitrile, 3-methyl-	ND	0	1.95 ± 0.01	0.23	ND	0
	Oxepine, 2,7-dimethyl-	ND	0	0.87 ± 0.04	0.1	ND	0
	Furfural	ND	0	ND	0	6.92 ± 0.13	0.66
	Ethyl tridecanoate	ND	0	ND	0	8.17 ± 0.02	0.78
	Phenol	ND	0	ND	0	0.49 ± 0.02	0.05
	Methyl tetradecanoate	ND	0	ND	0	0.54 ± 0.02	0.05
	Bicyclo[3.1.1]hept-2-ene-2-methanol, 6,6-dimethyl-	ND	0	ND	0	1.26 ± 0.05	0.12
	2-Ethylhexyl salicylate	ND	0	ND	0	1.31 ± 0.03	0.12
	**subtotal**	**36.820377**	**3.13**	**44.435835**	**5.23**	**38.626639**	**3.67**

* ND: not detected.
